# Supplementary material for: The Bowel-Associated Arthritis–Dermatosis Syndrome (BADAS): A Systematic Review
Source: Metabolites. 2023 Jun 25;13(7):790. doi: 10.3390/metabo13070790 (PMC10386568; doi:10.3390/metabo13070790)
Supplement: Supplementary file 1 [file metabolites-13-00790-s001.zip › metabolites-2376788-supplementary.pdf]

Supplementary Table S1. All included publications presenting cases of patients with bowel-associated dermatosis-arthritis syndrome

Year of publication	First author
1978	O'Loughlin S[4]
1979	Dicken CH[5]
1980	Morrison JG[6]
1980	Utsinger PD[7]
1981	Stein HB[3]
1981	Kennedy C[8]
1982	Fagan EA[9]
1983	Jorizzo JL[10]
1983	Fenske NA[11]
1983	Heyn J[12]
1984	Sandbank M[13]
1984	Dicken CH[14]
1985	Klinkhoff AV[15]
1985	Callen JP [16]
1986	Dicken CH[17]
1989	Delaney TA[18]
1990	Kemp DR[19]
1991	Cantatore FP [20]
1993	Varma JR[21]
1996	Matheson BK[22]
2003	Cox NH[23]
2003	Vázquez J[24]
2003	Mendoza JL[25]
2004	Slater GH[26]
2004	Brouard MC[27]
2005	Havemann BD[28]
2006	Guerre-Schmidt AR[29]
2006	Kawakami A[30]
2007	Ashok D[31]
2007	Prpic-Massari L[32]
2009	Patton T[33]
2009	Bouma G[34]
2011	Kinyó A[35]
2011	Lacey BW[36]
2011	Tu J[37]
2013	Truchuelo MT[38]
2014	DeFilippis EM[39]
2014	Pereira E[40]
2016	Aounallah A[41]

2016	Oldfield CW[42]
2016	Barland A[43]
2016	Zhao H[44]
2018	Rosen JD[45]
2018	Herbert J[46]
2019	Hassold N[47]
2020	Heard M[48]
2021	Havele SA[49]
2021	Caposiena Caro RD[50]
2021	Richarz NA[51]
2022	Johns HR[52]
2022	Alshahrani KS[53]

Supplementary Table S2. Cases excluded.

Year of publication	Authors	Reason*
1971	Shagrin JW [1]	1
1984	Jorizzo JL [98]	2
1998	Sarkany RP [99]	3
1998	Warin AP [100]	3
2013	Carubbi F [56]	4

*

1) Absence of cutaneous involvement
2) Already published cases
3) Alternative final diagnosis (Sweet syndrome, pyoderma gangrenosum)
4) Review without new patients

References included cases.

1) Shagrin, J.W.; Frame, B.; Duncan, H. Polyarthritis in obese patients with intestinal bypass. Ann. Intern. Med. 1971, 75, 377–380.
3) Stein, H.B.; Schlappner, O.L.; Boyko, W.; Gourlay, R.H.; Reeve, C.E. The intestinal bypass: Arthritis–dermatitis syndrome. *Arthritis Rheum*. **1981**, *24*, 684–690.
4) O'Loughlin, S.; Perry, H.O. A diffuse pustular eruption associated with ulcerative colitis. Arch Dermatol. 1978, 114, 1061-1064.
5) Dicken, C.H.; Seehafer, J.R. Bowel bypass syndrome. Arch Dermatol. 1979, 115, 837-839.
6) Morrison, J.G.; Fourie, E.D. A distinctive skin eruption following small-bowel by-pass surgery. Br J Dermatol. 1980, 102, 467-471.
7) Utsinger, P.D. Systemic immune complex disease following intestinal bypass surgery: bypass disease. J Am Acad Dermatol. 1980, 2, 488-495.
8) Kennedy, C. The spectrum of inflammatory skin disease following jejuno-ileal bypass for morbid obesity. Br J Dermatol. 1981, 105,425-435.
9) Fagan, E.A.; Elkon, K.B.; Griffin, G.F.; Kennedy, C.; Blenkharn, J.I.; Chadwick, V.S.; Hughes, G.R.; Hodgson, H.J. Systemic inflammatory complications following jejuno-ileal bypass. Q J Med. 1982, 51, 445-460.

10) Jorizzo, J.L.; Apisarnthanarax, P.; Subrt, P.; Hebert, A.A., Henry, J.C., Raimer, S.S., Dinehart, S.M., Reinarz, J.A. Bowel-bypass syndrome without bowel bypass. Bowel-associated dermatosis–arthritis syndrome. Arch. Intern. Med. 1983, 143, 457–461.
11) Fenske, N.A.; Gern, J.E.; Pierce, D.; Vasey, F.B. Vesiculopustular eruption of ulcerative colitis. Arch Dermatol. 1983, 119, 664–669.
12) Heyn, J.; Hey, H.; Jans, H.; Baek, L.; Ullman, S.; Halberg, P. Episodic arthritis, skin manifestations and immune complexes following intestinal by-pass operations for morbid obesity. Scand J Rheumatol. 1983, 12, 257-259.
13) Sandbank, M.; Weltfriend, S.; Wolf, R. Bowel bypass arthritis dermatitis syndrome: a histological and electron microscopical study. Acta Derm Venereol. 1984, 64, 79–82.
14) Dicken, C.H. Bowel-associated dermatosis-arthritis syndrome: bowel bypass syndrome without bowel bypass. Mayo Clin Proc. 1984, 59, 43-46.
15) Klinkhoff, A.V.; Stein, H.B.; Schlappner, O.L.; Boyko, W.B. Postgastrectomy blind loop syndrome and the arthritis-dermatitis syndrome. Arthritis Rheum. 1985, 28, 214–217.
16) Callen, J.P.; Woo, T.Y. Vesiculopustular eruption in a patient with ulcerative colitis. Pyoderma gangrenosum. Arch Dermatol. 1985, 121, 399–402.
17) Dicken, C.H. Bowel-associated dermatosis-arthritis syndrome: bowel bypass syndrome without bowel bypass. J Am Acad Dermatol. 1986, 14, 792-6.
18) Delaney, T.A.; Clay, C.D.; Randell, P.L. The Bowel-associated derma-tosis: arthritis syndrome. Australas J Dermatol. 1989, 30, 23–7.
19) Kemp, D.R.; Gin, D. Bowel-associated dermatosis–arthritis syndrome. Med. J. Aust. 1990, 152, 43–45.
20) Cantatore, F.P.; Carrozzo, M.; Loperfido, M.C. Post biliopancreatic bypass arthritis. Dermatitis syndrome. Clin Rheumatol. 1991, 10, 449–51.
21) Varma, J.R.; Pye, J.; Sinha, A. Dermal complication of the bowel bypass syndrome: a case report. J Fam Pract. 1993, 36, 564–566.
22) Matheson, B.K.; Gilbertson, E.O.; Eichenfield, L.F. Vesiculopustulareruption of Crohn's disease. Pediatr Dermatol. 1996, 13, 127–130.
23) Cox, N.H.; Palmer, J.G. Bowel-associated dermatitis–arthritis syndrome associated with ileo–anal pouch anastomosis, and treatment with mycophenolate mofetil. Br. J. Dermatol. 2003, 149, 1296–1297.
24) Vázquez, J.; Almagro, M.; del Pozo, J. Fonseca, E. Neutrophilic pustulosis and ulcerative colitis. J Eur Acad Dermatol Venereol. 2003, 17, 77–79.
25) Mendoza, J.L.; García-Paredes, J.; Peña, A.S. Cruz-Santamaría, D.M.; Iglesias, C.; Díaz Rubio, M. A continuous spectrum of neutrophilic dermatoses in Crohn's disease. Rev Esp Enferm Dig. 2003, 95, 233–236, 229–232. English, Spanish.
26) Slater, G.H.; Kerlin, P.; Georghiou, P.R.; Fielding, G.A. Bowel-associated dermatosis–arthritis syndrome after biliopancreatic diversion. Obes. Surg. 2004, 14, 133–135.
27) Brouard, M.C.; Chavaz, P.; Borradori, L. Acute pustulosis of the legs in diverticulitis with sigmoid stenosis: an overlap between bowel-associated dermatosis–arthritis syndrome and pustular pyoderma gangrenosum. J. Eur. Acad. Dermatol. Venereol. 2004, 18, 89–92.
28) Havemann, B.D.; Goodgame, R. A pustular skin rash in a woman with 2 weeks of diarrhea. MedGenMed. 2005, 7, 11.
29) Guerre-Schmidt, A.R.; Pelletier, F.; Carbonnel, F.; Conrad, M.A.; Perman, M.J.; Rubin, A.I.; Treat, J.R.. Syndrome arthrocutané associé à une maladie de Crohn chez une adolescente. Rev Med Interne. 2006, 27, 874–877.
30) Kawakami, A.; Saga, K.; Hida, T.; Jimbow, K.; Takahashi, H. Fulminant bowel-associated dermatosis-arthritis syndrome that clinically showed necrotizing fasciitis-like severe skin and systemic manifestations. J Eur Acad Dermatol Venereol. 2006, 20, 751–753.
31) Ashok, D.; Kiely, P. Bowel associated dermatosis - arthritis syndrome: a case report. J Med Case Rep. 2007, 5, 81.
32) Prpic-Massari, L.; Kastelan, M.; Brajac, I.; Cabrijan, L.; Zamolo, G.; Massari, D. Bowel-associated dermatosis–arthritis syndrome in a patient with appendicitis. Med. Sci. Monit. 2007, 13, CS97–C100.
33) Patton, T.; Jukic, D.; Juhas, E. Atypical histopathology in bowel-associated dermatosis–arthritis syndrome: a case report. Dermatol. Online J. 2009, 15, 3.
34) Bouma, G.; Neve, S.; van Grieken, N.C.; Stoof, T.J. A Crohn's disease patient with pustules and fever. Gut. 2009, 58, 1537–1564.
35) Kinyó, A.; Farkas, K.; Molnár, T.; Németh, I.; Varga,E.; Kemény, L.; Gyulai, R. Bowel-associated dermatosis-arthritis syndrome — a relapsing,extremely rare extra-intestinal sign of acute flare-up in Crohn'sdisease. Eur J Dermatol. 2011, 21, 443–445.
36) Lacey, B.W.; You, D.; Speziale, A. Pustular skin eruption in a patient with ulcerative colitis. Clin Gastroenterol Hepatol. 2011, 9, e43–e44.
37) Tu, J.; Chan, J.J.; Yu, L.L. Bowel bypass syndrome/bowel-associated dermatosis arthritis syndrome post laparoscopic gastric bypass surgery. Australas J Dermatol. 2011, 52, e5–e7.
38) Truchuelo, M.T.; Alcántara, J.; Vano-Galván, S.; Jaén, P.; Moreno, C. Bowel-associated dermatosis-arthritis syndrome: another cutaneous manifestation of inflammatory intestinal disease. Int JDermatol. 2013, 52, 1596–1598.

39) DeFilippis, EM, Magro, C., Jorizzo, JL. Bowel-associated dermatosis-arthritis syndrome in a patient with ulcerativecolitis: an extra-intestinal manifestation of inflammatorybowel disease. Clin J Gastroenterol. 2014, 7, 410–413.
40) Pereira, E.; Estanqueiro, P.; Almeida, S.; Ferreira, R.; Tellechea, O.; Salgado, M. Bowel-associated dermatosis-arthritis syndrome in an adolescent with short bowel syndrome. J Clin Rheumatol. 2014, 20, 322–324.
41) Aounallah, A.; Zerriaa, S.; Ksiaa, M.; Jaziri, H.; Boussofara, L.; Ghariani, N.; Mokni, S.; Saidi, W.; Sriha, B.; Belajouza, C. et al. Syndrome arthro-cutané lors d'une rectocolite ulcéro-hémorragique : une manifestation extra-intestinale rare des maladies inflammatoires de l'intestin. Ann Dermatol Venereol. 2016, 143, 377–381.
42) Oldfield, C.W.; Heffernan-Stroud, L.A.; Buehler-Bota, T.S.; Williams, J.W. Bowel-associated dermatosis-arthritis syndrome (BADAS) in a pediatric patient. JAAD Case Rep. 2016, 2, 272–274.
43) Barland, A.; Blackmon, J.; Fraga, G.; Aires, D.; Chain, R. Bowel-associated dermatosis-arthritis syndrome in a patient with Crohn disease. Cutis. 2016, 97, E1–E3.
44) Zhao, H.; Zhao, L.; Shi, W.; Luo, H.; Duan, L.; You, Y.; Li, Y.; Zuo, X. Is it bowel-associated dermatosis-arthritis syndrome induced by small intestinal bacteria overgrowth?. Springerplus. 2016, 5, 1551.
45) Rosen, J.D.; Stojadinovic, O.; McBride, J.D.; Rico, R.; Cho-Vega, J.H.; Nichols, A.J. Bowel-associated dermatosis-arthritis syndrome (BADAS) in a patient with cystic fibrosis. JAAD Case Rep. 2018, 5, 37–39.
46) Herbert, J.; Fox, C.; Khurshid, A. An interesting case of bowel-associated dermatitis arthritis syndrome. Rheumatology. 2018, 57(suppl_3), key075–228.
47) Hassold, N.; Jelin, G.; Palazzo, E.; Deschamps, L.; Forien, M.; Ottaviani, S.; Descamp, V.; Dieudé, P. Bowel-associated dermatosis arthritis syndrome: A case report with first positron emission tomography analysis. JAAD Case Rep. 2019, 5, 140–143.
48) Heard, M.; Zhang, M.; Jorizzo, J.L. A case of bowel-associated dermatosis-arthritis syndrome treated with ustekinumab: The importance of targeting underlying gastrointestinal disease. JAAD Case Rep. 2020, 6, 506–508.
49) Havele, S.A.; Clark, A.K.; Oboite, M.; Conrad, M.A.; Perman, M.J.; Rubin, A.I.; Treat, J.R. Bowel-associated dermatosis-arthritis syndrome in a child with very early onset inflammatory bowel disease. Pediatr Dermatol. 2021, 38, 697–698.
50) Caposiena Caro, R.D.; Di Prete, M.; Didona, D.; Orlandi, A.; Bianchi, L. Bowel-associated dermatosis arthritis syndrome and palisading neutrophilic granulomatous dermatitis as presentation of ulcerative colitis. Ital J Dermatol Venerol. 2021, 156(Suppl. 1 to No. 6), 7–8.
51) Richarz, N.A.; Bielsa, I.; Morillas, V.; Enguita, V.; Fumagalli, C. Bowel-associated dermatosis-arthritis syndrome (BADAS). Australas J Dermatol. 2021, 62, 241–242.
52) Johns, H.R.; Shetty, N.; Cash, J. .; Patel, T.; Pourciau, C. Bowel-Associated-Dermatosis-Arthritis Syndrome (BADAS) as early presentation of ulcerative colitis in an adolescent girl. Pediatr Dermatol. 2022, 39, 643–645.
53) Alshahrani, K.S.; Almohaya, A.M.; Hussein, R.S.; Ali, R.H. Enthesitis in Bowel-Associated Dermatosis-Arthritis Syndrome in an Ulcerative Colitis Patient. Case Rep Rheumatol. 2022, 4556250.
56) Carubbi, F.; Ruscitti, P.; Pantano, I.; Alvaro, S.; Benedetto, P.D.; Liakouli, V.; Giuliani, A.; Piccione, F.; Ciccia, F.; Amicucci, G. et al. Jejunoileal bypass as the main procedure in the onset of immune-related conditions: The model of BADAS. Expert. Rev. Clin. Immunol. 2013, 9, 441–452.
98) Jorizzo, J.L.; Schmalstieg, F.C.; Dinehart, S.M.; Daniels, J.C.; Cavallo, T.; Apisarnthanarax, P., Rudloff, H.B., Gonzalez, E.B. Bowel-associated dermatosis-arthritis syndrome: Immune complex-mediated vessel damage and increased neutrophil migration. Archives of internal medicine, 1984, 144, 738–740.
99) Sarkany, R.P.E.; Burrows, N.P.; Grant, J.W., Pye, R.J.; Norris, P.G. The pustular eruption of ulcerative colitis: a variant of Sweet's syndrome?. British Journal of Dermatology, 1998, 138, 365–366.
100) Warin, A.P. The pustular eruption of ulcerative colitis. Br J Dermatol, 1998, 139, 758–758.

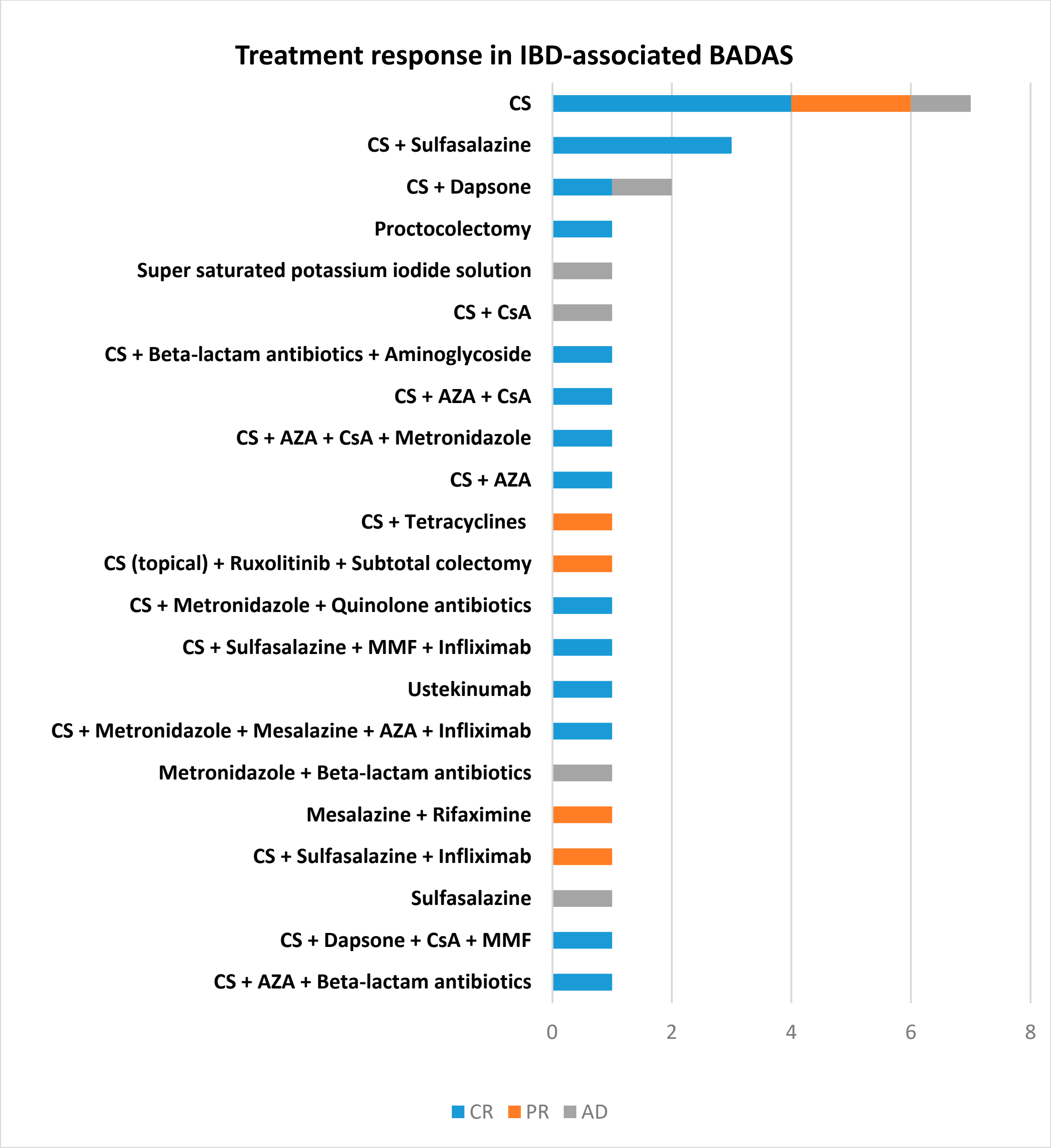

Treatment response in IBD-associated BADAS
CS
CS + Sulfasalazine
CS + Dapsone
Proctocolectomy
Super saturated potassium iodide solution
CS + CsA
CS + Beta-lactam antibiotics + Aminoglycoside
CS + AZA + CsA
CS + AZA + CsA + Metronidazole
CS + AZA
CS + Tetracyclines
CS (topical) + Ruxolitinib + Subtotal colectomy
CS + Metronidazole + Quinolone antibiotics
CS + Sulfasalazine + MMF + Infliximab
Ustekinumab
CS + Metronidazole + Mesalazine + AZA + Infliximab
Metronidazole + Beta-lactam antibiotics
Mesalazine + Rifaximine
CS + Sulfasalazine + Infliximab
Sulfasalazine
CS + Dapsone + CsA + MMF
CS + AZA + Beta-lactam antibiotics
0
2
4
6
8
CR
PR
AD

Supplementary Figure S1. Treatment response in IBD-associated BADAS. CR= complete remission; PR= partial remission; AD= active disease. CS= corticosteroid; CsA= Cyclosporine A; MMF = Mycophenolate mofetil; AZA= azathioprine.

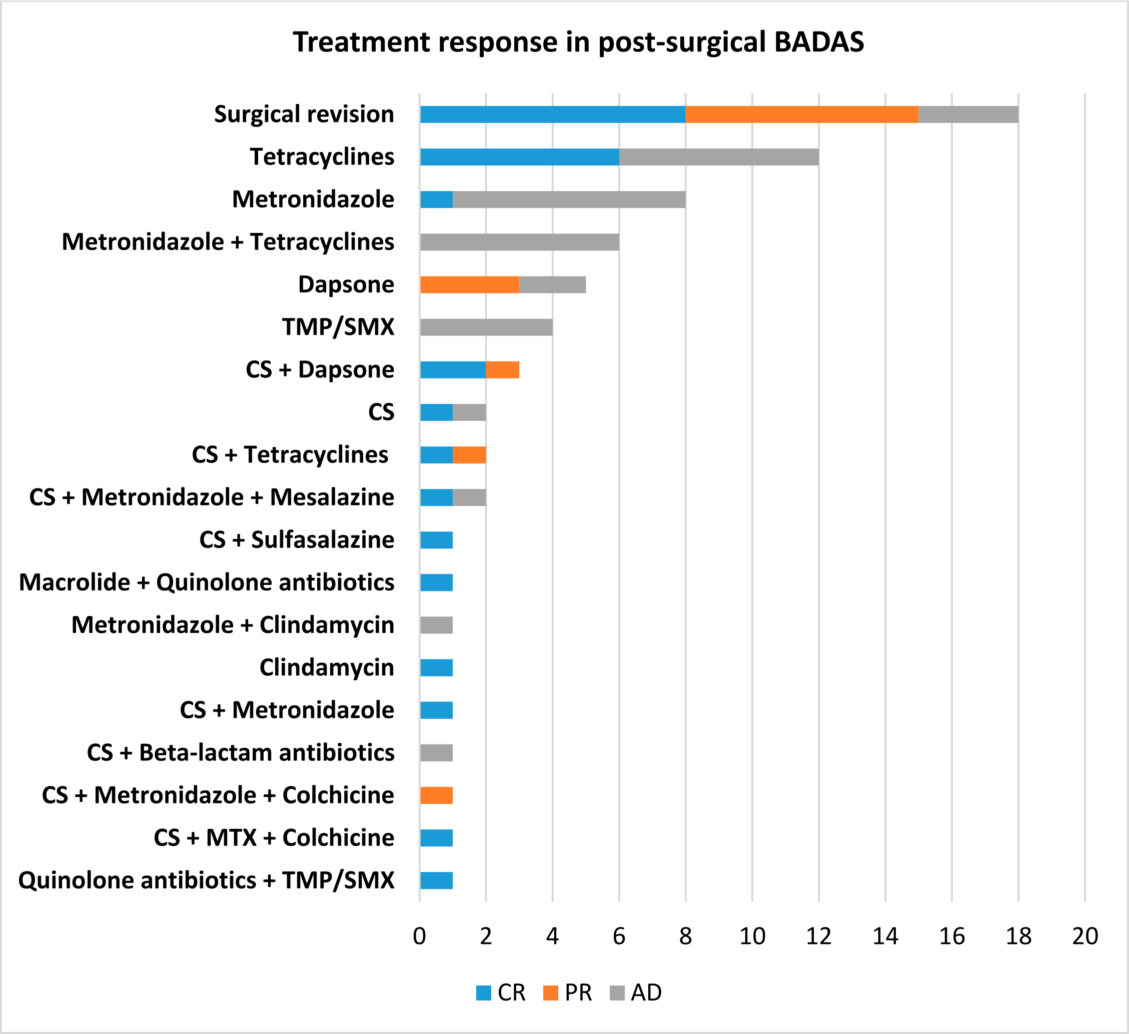

Supplementary Figure S2. Treatment response in post-surgical BADAS. CR= complete remission; PR= partial remission; AD= active disease. CS= corticosteroid; TMP/SMX= Trimethoprim / Sulfamethoxazole; MTX= methotrexate.
